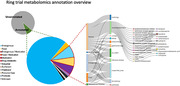# Metabolomics approaches for capturing the chemical exposome and its influences on cognitive function and brain health

**DOI:** 10.1002/alz.089874

**Published:** 2025-01-09

**Authors:** M Arthur Moseley, Oliver Fiehn, Pieter Dorrestein, Tuulia Hyotylainen, Matej Oresic, David Wishart, Nina Zhao, Rima F. Kaddurah‐Daouk, Alzheimer's Gut Microbiome Project Consortium (AGMP)

**Affiliations:** ^1^ Duke University, Durham, NC USA; ^2^ NIH‐West Coast Metabolomics Center, University of California, Davis, Davis, CA USA; ^3^ University of California, San Diego, La Jolla, CA USA; ^4^ Orebro University, Örebro Sweden; ^5^ Örebro University, Örebro Sweden; ^6^ University of Alberta, Edmonton, AB Canada; ^7^ University of California ‐ San Diego, La Jolla, CA USA

## Abstract

**Background:**

There is growing interest in the role of environmental factors (i.e., exposome) in the pathogenesis of Alzheimer’s diseases. The exposome includes three categories: internal (e.g., metabolism, gut microbiome, inflammation), specific external (e.g., environmental pollutants, diet, drugs, occupational), and general external (e.g., socioeconomic status, education, climate). The metabolome provides a readout of the influences of the exposome, capturing the presence of a large number of chemical exposures and allowing an interrogation of the influences of these chemicals on cognition and brain imaging changes.

**Method:**

Four centers of excellence in metabolomics have used mass spectrometry‐based capabilities to provide broad coverage of the chemical exposome. A ‘ring trial’ was performed to determine the coverage of the metabolome/exposome using state‐of‐the‐art analytical tools. Human serum/plasma standards from diseased individuals ‐ Alzheimer’s, COPD, IBS, osteoarthritis, and Type 2 diabetes (BioIVT), and NIST standards (1950 and 1958) were analyzed, using LC‐MS/MS (targeted and untargeted), Direct Flow‐MS/MS and/or ICP‐MS. Similarly, we are measuring the exposome/metabolome in large studies of AD patients, including ADNI, ADRCs and the ROSMAP brain collection.

**Result:**

Compounds were classified by chemical class (ClassyFire), toxicity/source groups (EPA CompTox DB)), and disease association (Comparative Toxicogenomics DB). Using the available InChiKey and CAS numbers available, ClassyFire sorted compounds into 184 chemical classes, EPA CompTox DB sorted 1179 unique InChiKey descriptors into 93 groups (toxicity class and/or exposure source), including DrugBank, Hazardous Substances DB 2019, BLOOD Toxic Substances Control Act, COSMO cosmetics and FDA Food Substances. These included 7 neurological groups with over 100 ring trial compounds. These compounds were classified as endogenous compounds, foods, medications, industrial chemicals, surfactants, plasticizers, personal care, and pesticides (Figure 1). Identical studies are being performed using large cohorts of brain and blood samples, including from ADRCs and the ROSMAP collection. We will present highlights of the brain chemical exposome and its links to cognition.

**Conclusion:**

Big data is being generated to capture the influences of the exposome on brain health, connecting these peripheral influences on brain metabolic health and disease, facilitating the development of novel therapeutic approaches targeting the exposome and its effect on brain health.